# The Professional Quality of Life of Domestic and Sexual Violence Advocates: A Systematic Review of Possible Risk and Protective Factors

**DOI:** 10.1177/15248380231171187

**Published:** 2023-05-18

**Authors:** Harriet Bromley, Sarah K. Davis, Blaire Morgan, Holly Taylor-Dunn

**Affiliations:** 1 University of Worcester, UK; 2 The Open University, Milton Keynes, UK

**Keywords:** systematic review, domestic and sexual violence advocates, risk factors, protective factors, ProQOL, compassion fatigue, compassion satisfaction

## Abstract

Professionals employed within the field of domestic and sexual violence (DV/SV) are known to experience both positive and negative psychological impacts because of the nature of their work. This review aims to establish which factors influence the professional quality of life (ProQOL) of DV/SV advocates. This group is known to face challenges that are specific to their working practices including scarce resources and frequent exposure to traumatic material. The systematic review protocol was designed based upon Preferred Reporting Items for Systematic Reviews and Meta-Analyses (PRISMA) 2020 guidance. Following a mixed-methods convergent segregated approach, a systematic search for qualitative and quantitative research within PsycINFO, Academic Search Complete, CINAHL, MEDLINE, Sage, Taylor & Francis, Wiley Online Library, and BASE was undertaken. Peer-reviewed empirical research and relevant gray literature, published in English, were considered for inclusion. Thirty articles were identified (16 quantitative, 13 qualitative, and 1 mixed-methods study), and assessed for methodological quality and risk of bias using established quality appraisal tools. An array of risk and protective factors emerged including communication competence, support from co-workers, office resources, and occupational stigma. A gap in the current evidence base was identified regarding the role that personal strengths may play in the well-being of those employed within the DV/SV sector. The ProQOL of DV/SV advocates is complex and dependent upon a variety of factors specific to their situation at the time. However, the findings of this review provide an important evidence base for future research avenues as well as policies and procedures for this workforce specifically.

## Introduction

The purpose of this review is to understand how advocates working with victim/survivors of domestic and sexual violence (DV/SV) can be better supported to perform their roles effectively, despite the occupational stressors they face. To achieve this, a systematic review of the literature was undertaken to develop a comprehensive understanding of the role that risk and protective factors play in the well-being of this workforce specifically. Within this review, the psychological factors associated with professional quality of life (ProQOL) are grouped as: (a) risk factors (that increase an individual’s vulnerability to experiencing negative outcomes) or (b) protective factors (that enhance adaptation and reduce the effects of stressful life events) (Lee et al., 2013).

A variety of DV/SV roles exist, with job titles and responsibilities varying across countries and organizations. Much of the existing research is based within the United States, yet differences are evident between the United Kingdom and US with divergent terms such as helpline or hotline staff and refuge or shelter staff. Many services aim to address both DV/SV; however, there are specialist roles for each. Within the United Kingdom, Independent Domestic Violence Advisors and Independent Sexual Violence Advisors exist, yet there are other roles associated with the DV/SV sector including community outreach workers and those based within sexual assault/rape crisis centers. For the purposes of this review, the above roles will be collectively referred to as DV/SV advocates.

These trained professionals deliver a range of support and advocacy services including the completion of risk assessments and safety planning, support in relation to criminal court cases and civil justice options, and facilitating access to target hardening or refuge accommodation (Howarth et al., 2009). In addition to this, advocates are also recognized as playing a vital role in multi-agency initiatives such as Multi-Agency Risk Assessment Conferences (Steel et al., 2011). They provide information and guidance on other services that victim/survivors may require including legal services, social services, health and social care, housing and benefits (Howarth et al., 2009). As such, they are required to liaise with a wide range of professions to include police officers, nurses, and social workers. Conflicts can often arise within these relationships due to the varied focus of these agencies and their approaches to victim/survivors of abuse (Behounek, 2011).

DV/SV advocates face a variety of stressors which exist at multiple levels of influence. This includes frequent exposure to detailed accounts of abuse suffered by DV/SV victim/survivors (Mihelicova et al., 2019). A wealth of research has demonstrated the negative impact that indirect exposure to DV/SV can have on those working within the sector (Brend et al., 2019; Crivatu et al., 2021). These range from physical symptoms such as headaches and fatigue, to emotional symptoms such as a heightened level of fear and hopelessness (Bishop & Schmidt, 2011; Clemans, 2004; Wasco & Campbell, 2002). In addition to this, many experience changes in their world view and issues within their own interpersonal relationships (Jirek, 2015; Rizkalla et al., 2021; Taylor et al., 2019). Such changes include feeling less safe in the world (Ben-Porat & Itzhaky, 2009), experiencing a diminished trust in others (Taylor et al., 2019) and increased feelings of vulnerability (Clemans, 2004). Those working with victim/survivors of sexual abuse have reported greater changes to their cognitive schemas than those working with cancer patients, suggesting human-induced trauma may be more difficult to process in comparison to that which occurs naturally (Cunningham, 2003). Risk and protective factors for the DV/SV workforce may, therefore, vary in comparison to other front-line support roles which also involve exposure to trauma.

Systematic reviews have been undertaken to collate the range of risk and protective factors shown to influence the well-being of other professions such as emergency responders (Kyron et al., 2021; Giménez Lozano et al., 2021) and child protection professionals (Molnar et al., 2020). Findings across these reviews highlight factors such as social support, workload, supervision quality, and coping strategies as playing a key role in the well-being of these professionals. While these factors may also influence DV/SV advocates, there is the potential for other, more specific, factors to influence the well-being of this workforce. Specialist DV/SV organizations arose from the feminist movement (Hague, 2021) and many advocates are survivors of DV/SV themselves (Slattery & Goodman, 2009). Additionally, research has shown that DV/SV advocates face stressors which are specific to this workforce, for example, accepting clients going back to abusive situations and enforcing rules in a refuge/shelter context (Merchant & Whiting, 2015).

Reviews conducted to date, typically include a focus on posttraumatic stress disorder, burnout, and secondary traumatic stress (STS). Within the DV/SV literature, compassion fatigue (CF) has been identified as a significant issue among advocates (Baird & Jenkins, 2003). CF is described as a stress reaction resulting from helping another individual who is suffering or traumatized (Figley, 1995) and can be considered an umbrella construct, drawing together burnout and STS as its core components. STS is defined as “the natural consequent behaviours and emotions resulting from knowing about a traumatising event experienced by a significant other – The stress resulting from helping or wanting to help a traumatized or suffering person” (Figley, 1995, p. 7).

Despite this, however, advocates also have a high likelihood of experiencing compassion satisfaction (CS) (Frey et al., 2017) which refers to the pleasure one derives from helping others and performing their role well (Stamm, 2010). ProQOL is a term used to encompass both the positive (CS) and negative (CF) impacts of exposure to trauma in the workplace (Stamm, 2010). This review takes a comprehensive approach by including all ProQOL-related constructs (i.e., CF, CS, burnout, or STS) as indicators of DV/SV advocate well-being. The review further includes vicarious trauma (VT) and vicarious resilience (VR), terms which are often used interchangeably with CF and CS within the literature (e.g., Meadors et al., 2010; Silveira & Boyer, 2015). VT is defined as the negative transformation in the helper that results from empathic engagement with trauma survivors and their trauma material, combined with a commitment or responsibility to help them (Pearlman & Caringi, 2009). In contrast, VR refers to the positive impact on and personal growth of therapists resulting from exposure to clients’ resilience (Hernández et al., 2007).

As a result of the negative impacts highlighted above, the DV/SV sector faces significant challenges regarding staff turnover (Merchant & Whiting, 2015). This issue can have far-reaching financial implications for organizations in the form of recruitment and training, as well as increasing pressure on remaining staff (Merchant & Whiting, 2015). These outcomes can lead to poorer quality advocacy services and ultimately, result in inconsistent support and unmet needs for clients. As such, it is important to develop a more comprehensive understanding of the role that both individual and environmental factors can play in the ProQOL of DV/SV advocates. It is also key to recognize that well-being in this workforce is complex due to the dynamic nature of many of these factors. Unlike static factors (such as a personal history of DV/SV), dynamic factors can change over time (e.g., levels of social support).

### The Present Review

While no systematic review exists for the DV/SV workforce, a scoping review for DV professionals (Brend et al., 2019) and a rapid evidence assessment review for SV professionals (Crivatu et al., 2021) have previously been undertaken. The key findings from these reviews include the significant impact that DV/SV work can have on the professional (both positive and negative), and the factors which influence their ability to cope with said impacts. Similar factors were identified across these reviews including the importance of workplace social support (WSS) from managers and supervisors, as well as informal support from peers. Both also highlighted the potential for a personal history of trauma to act as both a risk and protective factor.

While useful, these reviews adopted a wide scope by exploring a variety of roles including those that exist external to the specialist DV/SV sector such as social workers, counselors, police, and medical professionals. They also included professionals working with children (Crivatu et al., 2021) and perpetrators (Brend et al., 2019). Research has shown that working with these populations can have different impacts (Bozga et al., 2020; Voth Schrag et al., 2021) and their inclusion could therefore weaken the clarity of insights which relate specifically to professionals working with adult victim/survivors of DV/SV. Finally, neither of these reviews included a search of gray literature meaning important contributions which exist outside of academic databases may have been missed.

The current study will build upon these two previous studies by conducting a thorough systematic review which focuses specifically on those who provide advocacy services to adult victim/survivors of DV/SV within specialist third-sector organizations. These organizations are separate from those which exist within the public or private sector and include charities and voluntary groups which aim to deliver essential services to help improve people’s well-being. Specialist third-sector DV/SV services include safe houses or refuges, advocacy, outreach support, counseling, and helplines.

### Aims and Research Questions

To date, no systematic review of the risk and protective factors associated with the ProQOL of DV/SV advocates has been conducted. Within this review DV and SV will be considered alongside one another due to the significant overlap between the two sectors (Baird & Jenkins, 2003). The aim of this work is to systematically review the literature in order to develop a comprehensive understanding of the role that risk and protective factors play in the well-being of DV/SV advocates. Preferred Reporting Items for Systematic Reviews and Meta-Analyses (PRISMA) guidelines were followed with a protocol created prior to the start of the study. A mixed-methods approach^1^ was deemed most appropriate for this review because it provides a more comprehensive understanding of the evidence base than that offered by single method reviews (Stern et al., 2020). It enables the reviewer to triangulate findings between the quantitative and qualitative literature and to identify gaps in the current evidence base, thereby maximizing the usefulness of the review for informing future research and policy decision-making (Stern et al., 2020).

Given the mixed-methods approach adopted, two separate research questions will be used to guide the quantitative and qualitative elements of this review respectively:

1. Which factors are significantly associated with the ProQOL of DV/SV advocates and what is the nature of this relationship?
2. Which factors do DV/SV advocates perceive as being important for their ProQOL?

## Method

### Search Strategy

A systematic review was undertaken following PRISMA guidelines (Page et al., 2021). A mixed-methods convergent segregated approach was adopted, meaning the quantitative and qualitative data were independently synthesized and the subsequent quantitative and qualitative evidence was then integrated together (Stern et al., 2020). PsycINFO, Academic Search Complete, CINAHL, MEDLINE, SAGE, Taylor & Francis, and Wiley Online Library were searched in September 2021. While no date restrictions were applied, the results were limited to studies available in the English language and those that had been peer-reviewed. All empirical studies were included; while theoretical research, reviews, or books were excluded.

The key words chosen were based upon a review of literature in the area and followed a similar search strategy to that adopted by Crivatu et al. (2021). The words were separated into two categories where professional roles/work settings within the DV/SV field were combined with terms related to ProQOL using the word “AND.” Please refer to Supplemental Table A1 for a full list of the search terms.

Other relevant sources within the gray literature were also searched using the Bielefeld Academic Search Engine (BASE) which includes dissertations, theses, conference proceedings, and reports. The terms “domestic violence” OR “domestic abuse” and “sexual violence” OR “sexual abuse” were combined with three key ProQOL terms “burnout”, “compassion fatigue,” and “compassion satisfaction” using the word AND in six separate searches. A search of the job roles “Independent sexual violence*” and “Independent domestic violence*” were also undertaken. These key words were chosen as they were deemed to be the primary focus of the current review and therefore the most appropriate terms to identify gray literature relevant to the research questions. All articles published in English by September 2021 were included.

### Eligibility Criteria

The inclusion and exclusion criteria used to assess whether studies were eligible for inclusion within this review are discussed below, including the rationale for each.

(a) The sample population predominantly comprised professionals providing advocacy services to adult victim/survivors of domestic and/or sexual violence from within a specialist DV/SV organization or setting.

Studies were excluded if the sample predominantly comprised those providing DV/SV services to children or perpetrators. This decision was made due to differences associated with the impact of working with these groups. Research has shown that direct practice with survivors, but not offenders, had a significant relationship with CF (Voth Schrag et al., 2021) and those working with child victims of SV have described specific consequences of their work, to include changes in their relationships with their children (Bozga et al., 2020).

Studies were excluded if the participants worked with DV/SV victim/survivors; however, this was in a non-advocacy role or in a non-DV/SV-focused organization/setting. For example, mental health counselors providing therapeutic services, detectives investigating DV/SV offences within the police, or sexual assault nurse examiners working within an Emergency Department. Research has demonstrated that an array of differences exist between those providing DV/SV services in the third sector in comparison to those in the public or private sector. For example, the funding crisis faced by specialist third-sector DV/SV organizations means they experience additional barriers relating to inadequate funding, low pay, and scarce resources (Merchant & Whiting, 2015; Ullman & Townsend, 2007). A further difference is that specialist third-sector DV/SV organizations arose out of women’s activism, fighting for social change, and many are therefore based upon feminist principles (Hague, 2021). As such, a primary focus of these organizations is to raise awareness around DV/SV and participate in political action/system change efforts. Similarly, there may be differences as to the reasons why third sector and public/private sector staff have chosen to do the work that they do. Research has estimated that around 50% of advocates in the third sector are survivors of DV/SV themselves (Slattery & Goodman, 2009; Wood, 2017). Although figures such as this do not exist for those working in the public/private sector, there may be important differences in their motivation to do the work, and the subsequent impact this has on their well-being. Finally DV/SV advocates are independent from all agencies and, as such, focus on providing empowering, survivor-led support. This contrasts with other professionals working with DV/SV survivors in the public sector, for example, members of the police who are bound by statutory duties and investigative obligations.

(b) The sample population included paid members of staff.

Studies were excluded if the sample consisted entirely of volunteers.

Previous studies which included DV/SV advocates in both paid and voluntary positions found significant differences between the two samples (Babin et al., 2012; Baird & Jenkins, 2003). Of particular importance is the finding that staff experienced significantly higher levels of burnout in comparison to volunteers. The experiences of these two groups differ in several key areas, for example, the degree and type of client exposure, as well as the training and/or supervision they receive.

(c) The study explored coping strategies, risk factors or protective factors alongside their relationship with any of the ProQOL-related constructs (i.e., burnout, STS, CF, or CS).

Studies were excluded if they failed to explore the relationship between such factors and instead focused only on the positive or negative impacts of DV/SV advocates’ work. The rationale for this inclusion criteria was based solely around the research questions which guide this review. As stated previously, the impact of DV/SV advocacy work is already well-established (Clemans, 2004; Jirek, 2015; Wasco & Campbell, 2002), and as such, this review must add to knowledge by focusing on factors that have the potential to promote or reduce these impacts. In order to ensure that these criteria were met, quantitative papers were only selected if they explored the relationship between potential risk/protective factors and any ProQOL-related concept (i.e., the dependent variable was either CF, STS, burnout, VT, CS, or VR). Similarly, qualitative papers were only selected if they included discussion of advocates’ ProQOL (or well-being) alongside factors which had the potential to influence this.

### Study Selection

The study selection process was conducted in two parts. Firstly, databases were independently searched by HB (a PhD researcher), with titles and abstracts screened to fit the inclusion and exclusion criteria (further detail of which is provided below). Secondly, HB discussed study eligibility with SKD, BM, and HTD before narrowing the potential papers down for inclusion. There was a high level of agreement regarding which studies did and did not meet the eligibility criteria.

**Figure 1.**
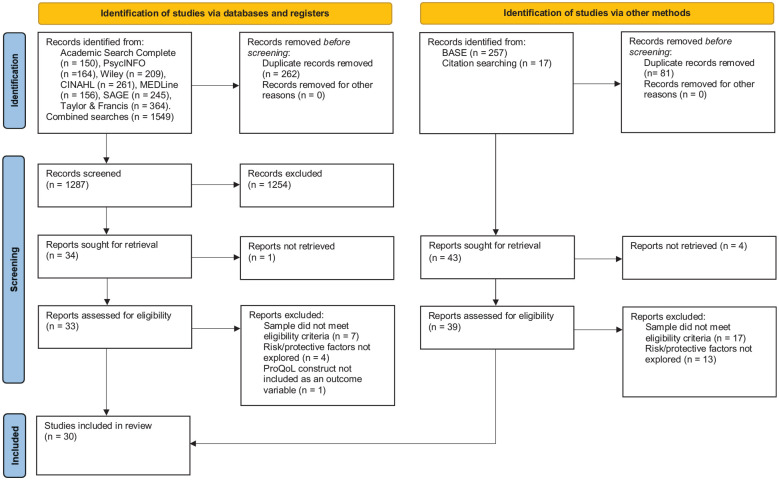
PRISMA 2020 flow diagram of search results. *Note.* PRISMA = Preferred Reporting Items for Systematic Reviews and Meta-Analyses.

Following the initial searches of the academic databases, a combined total of 1,549 articles were identified. Once duplicates were removed, 1,287 studies remained. Screening titles and abstracts resulted in the exclusion of 1,254 studies, leaving 34 studies to be read in full. Thirteen studies were excluded due to not meeting the eligibility criteria or the full text being unavailable. Reasons for exclusion include the sample population consisting only of volunteers, the sample population not providing advocacy services and a failure to explore risk or protective factors within the research. This resulted in a final sample of 21 published studies (see Figure 1).

Two hundred and fifty seven unpublished sources were identified using BASE. Once duplicates were removed, 176 articles remained. Following title and abstract screenings, 149 articles were excluded, leaving 27 articles to be read in full. Twenty one were excluded due to not meeting the eligibility criteria or the full text being unavailable (reasons for exclusion as above). This resulted in a total of six additional articles.

Manual searches of reference lists of related research resulted in the identification of a further three studies, meaning a total of 30 studies were included in the systematic review (See Figure 1 for PRISMA flow diagram).

### Data Extraction and Quality Assessment

Key study information was extracted, and all included studies were assessed for methodological quality and risk of bias using established quality appraisal tools. The Critical Appraisal Skills Programme (CASP; Critical Appraisal Skills Programme, 2018) was used for qualitative studies, and the Appraisal Tool for Cross-Sectional Studies (AXIS tool; Downes et al., 2016) for quantitative studies. Studies were coded for quality (either weak, moderate, or high).

### Risk of Bias Across Studies

According to the AXIS tool, eight of the quantitative studies received a moderate rating with the remaining nine studies receiving a high rating. According to the CASP tool, 1 of the qualitative studies received a weak rating, 2 a moderate rating, and 10 a high rating. Please refer to Supplemental Tables B1 and B2 for the global rating for each study.

## Results

When considering the characteristics of the included studies, 16 adopted a quantitative research design, 13 a qualitative research design, and 1 a mixed-methods research design (although only the quantitative phase of this study was included as the qualitative phase did not address the research question). Sample sizes ranged from 4 to 623 participants. Twenty two of the studies were undertaken in the United States, four in the United Kingdom, and one in each of the following countries: Canada, Ireland, Israel, and New Zealand. For details of the sample characteristics, ProQOL-related outcome variables and the measures used within each of the studies please refer to Supplemental Tables C1 and C2.

Findings indicated that an array of risk and protective factors exist. These are discussed in more detail below with the factors separated under the quantitative and qualitative research questions. While many of the studies addressed the research questions directly, others indirectly discussed aspects of well-being or coping within their overall aim. When describing the results below, a risk factor refers to a factor which was associated with higher levels of the negative impacts associated with ProQOL (i.e., CF, STS, and burnout). Similarly, a protective factor refers to a factor which was associated with higher levels of the positive impacts associated with ProQOL (i.e., CS or VR) or lower levels of the negative impacts. In addition to this, factors were further categorized under the following two subheadings: both risk and protective factors (for factors which were associated with both positive and negative impacts) and mixed effect factors (where the findings lacked consistency). Please refer to Table 1 for a summary of the risk and protective factors identified within the quantitative and qualitative literature.

**Table 1.** Summary Table Showing the Risk and Protective Factors Identified Across the Quantitative and Qualitative Studies.

Methodology	Identified Factors
Quantitative	Risk factors
	Younger age
	High levels of neuroticism
	High levels of communication anxiety
	Stronger feminist beliefs
	Low levels of self-efficacy for being productive at work
	Experiencing recent life stress
	Higher hours of advocacy work per week
	High levels of co-worker stress
	Working with outside agencies
	After-hour responsibilities
	High levels of time pressure
	Lack of office resources
	Low levels of control at work
	Unmanageable workloads
	Exposure to microaggressions within the workplace
	Higher hours spent per week in case management/staff meetings
	Higher hours of training prior to employment
	Misaligned values with the organization
	Negative occupational stigma
	Protective factors
	Older age
	Higher levels of education
	Stronger feminist beliefs
	High levels of belief in a just world
	High levels of communication competence
	High levels of self-efficacy for dealing with stressors at work
	Knowledge of the negative impacts associated with exposure to trauma
	The “good soldiering” experience
	Social support from family and friends
	Job satisfaction
	Shared power
	Job security
	A supportive organization which has a sense of community
	High levels of time pressure
	Receiving meaningful rewards
	High levels of control at work
	Manageable workloads
	Alignment between personal and organizational values
	Positive occupational stigma
	Mixed factors
	Length of experience in the DV/SV sector
	A personal history of trauma
	Client exposure/hours of direct contact with survivors
	Effective supervision
	WSS
	Perception of fairness within the organization
	The type and frequency of coping and self-care strategies deployed
Qualitative	Risk factors
	Out-of-hours work
	Lack of adequate pay
	Poor work conditions
	Scarce resources
	High workloads
	Time constraints
	Rigid work demands (inflexibility)
	Supervisors who failed to model effective self-care strategies
	Lack of or ineffective training
	Lack of or ineffective supervision
	Unhealthy organizational norms and collective expectations
	Organizations which fail to recognize or value survivors’ expertise
	A personal history of abuse
	Broader societal attitudes (i.e., rape myths or societal denial of rape)
	Collaboration issues with other professionals
	Protective factors
	Years of experience in the sector
	Higher levels of education
	The use of coping strategies (i.e., the ability to desensitize, be reflective, remain self-aware, and create emotional boundaries)
	Self-care practices
	Social support from partner/family/friends
	Organizations that encourage their staff to engage in self-care and have policies and procedures in place for staff well-being
	The provision of regular, high quality training
	The provision of regular, high quality supervision
	WSS
	An organization which values survivors’ expertise

*Note*. Total sample size across the quantitative studies = 3,368. Total number of scales used across the quantitative studies = 10. Total sample size across the qualitative studies = 280. DV/SV = domestic and sexual violence; WSS = workplace social support.

### Quantitative Findings

Which factors are significantly associated with the ProQOL of DV/SV advocates and what is the nature of this relationship?

#### Risk factors

Several factors specific to DV/SV advocates themselves were identified as potential risk factors. Advocates with higher levels of neuroticism (Flatter, 2000), higher levels of communication anxiety (Babin et al., 2012), and lower levels of self-efficacy for being productive at work (Baker et al., 2007) were more likely to experience the negative ProQOL impacts. The experience of recent life stress was also shown to be a risk factor for DV/SV advocates (Voth Schrag et al., 2021).

A number of work-related risk factors were identified in one study to include an advocate’s perceptions of their colleagues’ stress levels, working with outside agencies, after-hour responsibilities and a lack of office resources (Bemiller & Williams, 2011). Additional risk factors include exposure to microaggressions within the workplace (Voth Schrag et al., 2021), hours spent per week in case management/staff meetings (Flatter, 2000) and surprisingly, hours of training prior to employment (Ganz, 2015). A further consistent finding within the quantitative literature related to higher hours of advocacy work per week as a risk factor (Bemiller & Williams, 2011; Dekel & Peled, 2000; Ganz, 2015). Finally, an advocate’s perception of their physical safety at work was investigated as a potential risk factor by one study; however, no evidence was found to support this (Bemiller & Williams, 2011).

#### Protective factors

Knowledge of the negative impacts associated with exposure to trauma was shown to act as a protective factor for advocates (Campbell, 2008), as well as higher levels of education in general (Baird & Jenkins, 2003; Dekel & Peled, 2000). A further protective factor related to advocates who had high levels of belief in a just world (Horvath et al., 2021), a concept which asserts that good things tend to happen to good people and bad things to bad people (Lerner, 1980). One study identified the protective nature of the “good soldiering” experience which they defined as meshing individual motivation with the position of mission-type work (Bemiller & Williams, 2011). In addition to this, advocates with high levels of communication competence (Babin et al., 2012) and high levels of self-efficacy for dealing with stressors at work (Baker et al., 2007) were shown to be less likely to experience the negative ProQOL impacts.

Work-related factors shown to act as protective factors for advocates include high levels of job satisfaction (Flatter, 2000), shared power (Slattery & Goodman, 2009), and job security, as well as the opportunity to receive meaningful rewards (Wachter et al., 2020). In addition, a supportive organization which has a sense of community (Frey et al., 2017; Voth Schrag et al., 2021; Wachter et al., 2020), and social support from family and friends (Brown & O’Brien, 1998; Frey et al., 2017; Ganz, 2015) were both identified as key protective factors for this workforce.

#### Both risk and protective factors

Several factors were shown to act as both risk and protective factors depending on their direction. Firstly age, the cross-sectional literature consistently identified younger age as a risk factor (Baird & Jenkins, 2003; Dworkin et al., 2016; Flatter, 2000) and older age as a protective factor (Ganz, 2015; Voth Schrag et al., 2021) for the well-being of DV/SV advocates. In addition, an advocate’s perceptions of their workload, the level of control they have over their work and work environment, and whether they feel aligned with the values of their organization (Kulkarni et al., 2013; Voth Schrag et al., 2021; Wachter et al., 2020) were all identified as both risk and protective factors. Advocates who viewed these factors in a negative light (i.e., unmanageable workloads, low levels of control, and misaligned values) were more likely to experience negative impacts. In contrast, those who viewed these factors in a positive light (i.e., manageable workloads, high levels of control, and feeling aligned with their organization’s values) were more likely to experience positive impacts.

A complicated relationship was identified regarding time pressure (Baker et al., 2007), feminist beliefs (Fedele, 2018), and occupational stigma (Ganz, 2015), all of which were identified as acting as both risk and protective factors but in the same direction. While high levels of these factors increased the likelihood of advocates experiencing negative impacts, they also increased their likelihood of experiencing positive impacts.

#### Mixed effect factors

Mixed and inconsistent results were found with regards to several factors. Firstly, an advocate’s length of experience in the DV/SV sector was identified as a protective factor by several studies (Baird & Jenkins, 2003; Dekel & Peled, 2000; Frey et al., 2017; Ganz, 2015; Kulkarni et al., 2013), with only one study finding that more experienced advocates were at higher risk of experiencing negative impacts (Horvath et al., 2021). Similarly, whether an advocate has a personal history of trauma has been identified as both a risk factor (Dworkin et al., 2016; Slattery & Goodman, 2009) and a protective factor (Frey et al., 2017), with two studies finding no association (Bemiller & Williams, 2011; Voth Schrag et al., 2021). Regarding client exposure, working in a role that requires direct contact with survivors has been identified as a risk factor (Babin et al., 2012; Dekel & Peled, 2008; Voth Schrag et al., 2021), with higher client loads shown to increase risk at both an individual (Horvath et al., 2021) and an agency level (Dworkin et al., 2016). However, other studies have not supported this finding (Baird & Jenkins, 2003; Fedele, 2018; Slattery & Goodman, 2009).

Effective supervision has been identified as a protective factor (Brown & O’Brien, 1998; Campbell, 2008; Kulkarni et al., 2013; Slattery & Goodman, 2009; Voth Schrag et al., 2021); however, one study found no evidence of a relationship (Bemiller & Williams, 2011). WSS was shown to act as a protective factor (Campbell, 2008; Flatter, 2000; Slattery & Goodman, 2009), particularly informational WSS (Babin et al., 2012), yet other studies found no association (Baker et al., 2007; Bemiller & Williams, 2011; Dekel & Peled, 2000). An advocate’s perception of fairness within their organization was identified as a protective factor by two studies (Voth Schrag et al., 2021; Wachter et al., 2020), with a separate study finding no evidence to support this (Bemiller & Williams, 2011).

Finally, the type and frequency of coping and self-care strategies deployed was identified as a protective factor (Brown & O’Brien, 1998; Kulkarni et al., 2013; Wachter et al., 2020); this includes the use of professional boundaries (Ganz, 2015). However, certain coping strategies have been identified as risk factors (Horvath et al., 2021) to include spending time in leisure (Kulkarni et al., 2013) and mental disengagement (Brown & O’Brien, 1998). A separate study failed to find any relationship between coping/self-care strategies and ProQOL (Baker et al., 2007).

### Qualitative Findings

Which factors do DV/SV advocates perceive as being important for their ProQOL?

#### Risk factors

Within the qualitative literature, DV/SV advocates described an array of work-related factors which had a negative impact on their ProQOL. This includes undertaking out-of-hours work (Behounek, 2011; Massey et al., 2019), receiving inadequate pay (Behounek, 2011; Ullman & Townsend, 2007), poor work conditions, scarce resources (Kreinath, 2019), high workloads (Jirek, 2020; Massey et al., 2019), time constraints, rigid work demands, and inflexibility in the workplace (Ullman & Townsend, 2007).

DV/SV advocates with a personal history of abuse described this as negatively impacting their ProQOL, particularly due to their reluctance to share this information with their employers (Behounek, 2011). This was due to fear of being stigmatized as a victim and potentially seen as less effective in their role. Of a similar note, broader societal attitudes such as rape myths or societal denial of rape were identified as indirect risk factors for advocates in the sense that they created additional barriers to service provision (Ullman & Townsend, 2007). These attitudes were reflected in societal institutions’ reactions to victim/survivors and were manifested in system responses that ultimately interfered with advocacy. Finally, collaboration issues with other professionals were described as a risk factor by DV/SV advocates in one study (Behounek, 2011). This includes interactions with the police, medical professionals, and those employed within the criminal justice system who they described as “hostile collaborators.”

#### Protective factors

The use of coping strategies and self-care practices were consistently identified as playing an important role in the ProQOL of DV/SV advocates within the qualitative literature (Horvath et al., 2020; Kreinath, 2019; Massey et al., 2019; Taylor et al., 2019; Wasco et al., 2002). This includes organizations that encourage their staff to engage in self-care (Wilson & Goodman, 2021). Receiving social support from partners/family/friends was commonly referred to as an important strategy deployed by advocates to cope with the stressors associated with DV/SV work. Further coping strategies highlighted as important for advocates’ well-being include their ability to desensitize (Massey et al., 2019), be reflective (Horvath et al., 2020), remain self-aware, and create emotional boundaries (Molloy, 2019).

WSS from colleagues was consistently identified as a protective factor for this workforce (Brend & MacIntosh, 2021; Horvath et al., 2020; Kreinath, 2019; Massey et al., 2019; Wasco et al., 2002). These studies described how advocates would “organically” form “support groups” or “well-being initiatives” with their co-workers that would provide them with a source of support both inside and outside of work. One study found that for the support to be deemed effective, and therefore act as a protective factor, the individual providing it must possess particular skills. This includes effective emotion management skills and an ability to accept situations and the associated emotions (Brend & MacIntosh, 2021).

An advocate’s length of experience in the sector was described as a protective factor in two studies, with those who had worked in the sector for a longer period described as less likely to experience negative impacts (Kreinath, 2019; Molloy, 2019). One study also referred to an advocate’s level of education as a protective factor (Molloy, 2019).

#### Both risk and protective factors

A lack of training was identified as a risk factor by DV helpline staff who stated that the absence of training on the emotional impact of their work significantly challenged their well-being (Taylor et al., 2019). In contrast, staff based at a Sexual Assault Referral Centre highlighted that effective training is a protective factor as it has the potential to reduce the negative emotional responses typically associated with this line of work (Horvath et al., 2020).

In addition, poor quality supervision was described as a risk factor (Kreinath, 2019), with advocates in one study citing this as a key factor which affected their well-being and their subsequent decision to leave the sector (Ullman & Townsend, 2007). A further study identified a lack of regular supervision as a risk factor, with staff highlighting that consistency was essential in supporting the well-being of staff (Taylor et al., 2019). Related to this, advocates who felt their supervisors failed to model effective self-care strategies were deemed to be at increased risk of experiencing negative impacts (Jirek, 2020). In contrast, effective supervision was deemed to be a protective factor with DV/SV advocates describing this as an essential resource (Horvath et al., 2020) and extremely helpful (Massey et al., 2019) in enabling them to cope with the stressors associated with their line of work.

DV/SV advocates emphasized the importance of working for a supportive organization which has policies and procedures in place for staff well-being (Molloy, 2019; Wasco et al., 2002). In contrast, organizations which were not perceived as supportive were identified as increasing the risk of negative impacts (Ullman & Townsend, 2007), including those which lack management strategies to help alleviate stress (Behounek, 2011). Two qualitative case studies described unhealthy organizational norms and collective expectations as overarching risk factors for DV/SV advocates (Jirek, 2020; Jury et al., 2018). This includes expectations to work additional hours, accept additional workloads, and to not show any emotion in response to their work. It also includes the burden of self-care being placed on the individual as opposed to the organization and the well-being of clients being prioritized over that of staff. A separate study of DV shelter staff found that a supportive culture was an important protective factor, with those with less supportive cultures being more likely to leave the field (Merchant & Whiting, 2015).

Finally, one study found that an advocate’s views on whether they share values with their organization could act as both a risk and protective factor (Wilson & Goodman, 2021). This was specifically in relation to advocates who were survivors themselves, with participants describing being part of an organization which creates a sense of belonging and values their survivor expertize as being key to their well-being.

## Discussion

The aim of this systematic review was to summarize the literature regarding the role that risk and protective factors play on the ProQOL of DV/SV advocates. A complex array of factors were identified, although the consistency of these findings was mixed. Elements of the rapid evidence assessment and scoping reviews previously conducted in this field were supported, including the impact of high-quality training and supervision. Several new risk and protective factors emerged from this review, and although not all findings were consistent, they do offer new avenues for researchers to investigate. This includes the role of age, occupational stigma, communication skills, belief in a just world, exposure to microaggressions, and unhealthy organizational cultures.

Certain factors were supported across the quantitative and qualitative literature, for example, support from family and friends, a sense of community, and working with external agencies. However, contradictory findings across the quantitative and qualitative literature were also identified. A personal history of trauma was consistently highlighted as playing an important role in the qualitative literature yet produced mixed results in the quantitative literature. Similarly, effective WSS was consistently highlighted in the qualitative literature, yet mixed results were found in the quantitative literature. The quality and frequency of supervision was consistently highlighted as being imperative within the qualitative literature, however, this was not supported by all the quantitative studies.

Certain aspects of the qualitative evidence may help to explain differences identified in the quantitative studies. For instance, mixed results were found in the quantitative literature regarding WSS, yet this was consistently identified as a protective factor within the qualitative literature. The qualitative studies expanded upon the quantitative findings with advocates explaining that the provision of WSS is not always sufficient. For this to be deemed effective, the colleague providing it must possess particular skills (e.g., the ability to demonstrate effective emotion management). This offers a potential explanation as to the mixed results identified in the quantitative literature. Although the advocates in these studies may have received WSS from colleagues, this does not necessarily mean they deemed it to be effective, hence the reason why some studies may have failed to find a relationship between this factor and well-being.

Feminist beliefs (Fedele, 2018) and occupational stigma (Ganz, 2015) were shown to affect the well-being of DV/SV advocates in both a positive and a negative way. When interpreting the findings, it is possible that these two factors may be interlinked. Specialist DV/SV organizations arose from the feminist movement, with advocates providing survivor-centered support based upon feminist principles (Hague, 2021). It is this empowerment-based approach which sets advocates apart from other professionals (Wood, 2014; Wood et al., 2020). Advocates who endorse stronger feminist beliefs may have greater awareness of social inequities and therefore be more open about their job role and their hopes to promote social change. This includes a sense of duty around demystifying and de-stigmatizing gender-based violence through open disclosures. However, as a result, they may be more likely to experience negative occupational stereotypes (avoidance/hostility) when meeting new people and introducing their work identity. As such, they may be at increased risk of experiencing burnout.

Similarly, those with higher feminist beliefs who are willing to talk about their job role passionately may also be more likely to experience the “favorable” occupational stereotypes (idealization/encouragement/sympathy) associated with their job. These positive encounters when introducing their occupation can lead advocates to experience higher levels of external social support and subsequently a reduction in feelings of burnout. The above demonstrates how the same factors could lead advocates to experience both distress and growth simultaneously.

The occupational stigma faced by advocates is often based around the association between DV/SV work and feminism and the subsequent stereotypical view of feminists. For example, in one study advocates described being labeled as a “bunch of humorless man-haters” by strangers they encounter in everyday life (Ganz, 2015, p. 106). However, such occupational stigma can also be directed toward advocates by those working in partner agencies. For example, there are common misconceptions regarding the advocacy role, and many advocates describe collaboration issues with male police officers, judges, and lawyers due to the patriarchal nature of the criminal justice system (Behounek, 2011).

Several factors were investigated within the quantitative literature that were not mentioned within the qualitative literature. This includes the role of self-efficacy, belief in a just world and exposure to microaggressions. Of the qualitative studies, only one made brief reference to level of education and only two referred to years of experience. Yet these factors were consistently included as independent variables within the quantitative studies. This highlights the key methodological differences between the quantitative and qualitative literature. While the quantitative research is based upon the researcher’s interests and/or previous quantitative studies or theory, the qualitative research provides rich data on the respondents’ stories and perspectives. This disconnect offers further support for the usefulness of mixed-methods approaches.

One of the most consistent findings within the review related to age, with younger advocates being more likely to experience the negative impacts of the work in comparison to their older colleagues. It could be assumed that this relationship is due to older advocates having more experience working in the DV/SV sector. However, interestingly, one study demonstrated that the relationship between age and CF persisted even when controlling for years of experience (Voth Schrag et al., 2021). It has been suggested that older people are less prone to experiencing burnout in general as they are more likely to have a balanced perspective on life and are “older but wiser” (Maslach, 1982, p. 100).

Another key finding across the quantitative and qualitative literature relates to how a supportive organization which prioritizes well-being can act as an important protective factor. This includes organizations which “normalized” the fact advocates are at risk of experiencing negative impacts in response to their work and validated their subsequent feelings and emotions. It is possible that, as a result, advocates felt less stigma and were therefore less likely to hide their symptoms and more likely to open up and discuss them with a colleague or supervisor at an earlier stage. This then links into WSS and effective supervision which were emphasized as playing a key role in well-being, particularly throughout the qualitative literature.

### Limitations

Regarding the review process, there are a number of limitations which are common to all systematic reviews. Due to the restrictions placed on the eligibility criteria, that is, only articles published in English, it is possible that excluded studies could have provided important findings and results which would expand upon our understanding in this area. Secondly, due to the wide range of job titles/settings in the field as well as different terminology and spelling used across countries, it is possible that not all eligible studies were identified due to the key terms adopted. As most of the included studies were conducted in the United States, there is a risk of cultural bias making it difficult to assess how generalizable the findings are to other countries. It is possible that the key terms used were primarily relevant to advocates based within the United States or United Kingdom and therefore important studies from other countries were missed.

A further potential limitation relates to the inclusion/exclusion criteria used to assess the eligibility of the samples used in the chosen studies. DV/SV work encompasses a large variety of roles within a range of settings, all of which involve specific stressors and differing levels of exposure to trauma. As such, the researchers had to take steps to ensure that these were adequately captured within the key terms used to search the databases. Some studies chose to include DV/SV professionals from a range of professions/settings, whereas others included professionals working in a single role in a specific organization. As some of the studies included within this review failed to specify exact numbers/proportions in terms of job roles when numerous were included, it was difficult for the researcher to make accurate assessments on some occasions. This involved making judgement choices based upon a number of the studies which included mixed samples within the sector.

The inclusiveness of this review is a key strength given that published and gray literature articles were included with no time limits applied and both quantitative and qualitative designs. By synthesizing the findings from both quantitative and qualitative literature, this enables the reviewer to provide a richer understanding of the subject area (Stern et al., 2020).

### Directions for Future Research

A common thread of limitations was identified throughout the quantitative studies included in the review, many of which could be addressed in future research. Most notably, all the studies were cross-sectional in design, meaning that cause and effect cannot be inferred and the reasons why these factors may or may not be related cannot be determined. Secondly, a wide variety of factors were only investigated once within the quantitative studies and comparisons therefore cannot be made regarding the consistency of these findings. A wide variety of measures were deployed to assess the positive and negative impacts of DV/SV work (the dependent variables). While there is significant overlap between these concepts, the range of measures used makes it difficult to draw comparisons across studies. However, as the aim of the review was to provide an overview of the factors known to affect ProQOL, a broad approach to well-being was deemed essential. Additionally, many of the studies identified limitations associated with the reliability or validity of the measures they used (Baker et al., 2007; Campbell, 2008). Some studies acknowledged their failure to include, and control for, other important variables known to affect the well-being of DV/SV advocates within their analysis meaning we cannot be sure what role these factors did or did not play in relation to their overall findings (Fedele, 2018; Slattery & Goodman, 2009). Finally, some of the quantitative studies had particularly low participant numbers (less than 100) (Babin et al., 2012; Brown & O’Brien, 1998; Dekel & Peled, 2000) and the majority failed to provide a rationale for their sample size.

As previously stated, there are numerous inconsistencies across the quantitative results, and these must be considered in relation to the limitations highlighted above. The reader should consider the influence of design bias and sampling bias when interpreting the mixed findings. For example, the inconsistent results identified across many of the factors could be due to the varied measures and sample populations included within the quantitative studies. It would be beneficial for future research to use clearer, more consistent, terminology, definitions, and measures to assess well-being in this workforce.

The qualitative studies all recognized that their small sample sizes were a limitation in terms of the generalizability of their findings. They acknowledged that their findings may only be representative of the specific DV/SV organization, or specific geographic location that the study was based within. Readers were advised to draw caution before applying these findings to other organizations, or locations given that they are likely to differ in several key areas (staff members, policies and procedures, level of funding, etc.). However, it is important to acknowledge the difficulties associated with gaining access to specialist and highly confidential DV/SV organizations in the first place. A further limitation of the qualitative literature is that in most of the studies, the researcher/s failed to sufficiently cover their role and the potential for bias in terms of developing the study, influencing participants, and analyzing the data. This is particularly important given that some of the researchers acknowledged they were current or previous employees of the DV/SV sector or even the specific organization which they were studying (Jury et al., 2018).

Regarding the participants providing open and honest answers, the studies included within this review had the potential for social desirability bias. Although participants were informed that their results would not be shared with their organization, many may have feared this and subsequently answered in a way which would suggest that they are coping. Self-selection bias is also a concern as all participants volunteered to be in the studies. It is therefore possible that those who did volunteer felt that they had the time to participate (and therefore did not deem themselves as having an unmanageable workload for example). It is also possible that those who volunteered had strong feelings about their job or organization and were keen to share their either positive or negative views with the researcher. This method of recruitment across the board means that many important voices/results may not have been captured. For example, those suffering from severe burnout may have been signed off work with sickness and were therefore not included within the research. Similarly, only three of the studies included advocates who had already left the sector (Behounek, 2011; Merchant & Whiting, 2015; Ullman & Townsend, 2007). These participants would likely have important information to share.

A final limitation of the studies included within this review relates to the objectivity of many of the factors which were investigated. Participants were asked in both the quantitative and qualitative studies to provide their perceptions of factors such as their workload or the level of control they have at work. The answers they provided can be seen as subjective, given that this could vary significantly across participants. Objective assessments of these factors were not undertaken and this could be addressed in future research.

In terms of diversity, the large majority of participants identified as White females educated to degree level. Many of the studies included a 100% female sample with some stating that they chose to exclude male participants. This was recognized as a limitation in most studies; however, many argued that although the findings are not applicable to the general population, they are largely representative of the DV/SV workforce. For example, one of the case studies was based upon an all-female DV agency (Jirek, 2020) and additional research in the field supports these demographics as being representative (Wood, 2017). Despite this, future research in this area would benefit from utilizing more diverse samples (e.g., in terms of race and gender identity) to increase generalizability. This is particularly important given that race-related microaggressions were identified as a risk factor for advocates within this review (Voth Schrag et al., 2021). In addition, a particular focus on the experiences of advocates who belong to distinct social and cultural groups, such as those who identify as indigenous, would be beneficial.

While a similar number of quantitative and qualitative studies were identified in the process of this review, only one mixed-methods study was included, highlighting a potential gap which future research could focus on filling. It was also evident that the majority of cross-sectional studies focused on measuring the negative ProQOL impacts (i.e., burnout, CF, STS) as their dependent variable with only five including a measure of the positive ProQOL impacts (i.e., CS, VR). This suggests a need for a positive psychological approach where research focuses on which factors have the potential to promote the positive impact of the work, as well as buffer against the negative.

It is evident from the findings that the majority of studies examined organizational factors and whilst some individual level factors, such as age, were included, how these relate to psychological factors is under-explored. Reviews such as this are valuable in that they enable the identification of areas of interest which warrant further investigation. A gap in the current evidence base was identified regarding the role that personal strengths may play in the well-being of those employed within the DV/SV sector. Personal strengths have been shown to account for well-being over and above other resilience-related factors (Martínez-Martí & Ruch, 2017). Several strengths and their relationship with well-being have already been explored in other professions exposed to trauma. For example, in nurses, high levels of emotional intelligence have been linked with lower levels of burnout (Szczygiel & Mikolajczak, 2018). In the current review, some studies referred to the importance of emotion management skills for this workforce (Behounek, 2011; Molloy, 2019). Given the lack of detail provided on this factor, it may be beneficial for future research to explore the role of personal strengths, such as emotion management, on the well-being of DV/SV advocates.

As personal strengths are not fixed, there is the potential for organizations to use information such as this to inform and implement strengths-based interventions, policies, and procedures all aimed at fostering well-being. Previous research has shown that such interventions can be highly effective (Schutte & Malouff, 2019). By providing training to support particular personal strengths in DV/SV advocates, this is equipping them to manage their well-being, particularly when dealing with the emotional aspects/stressors of the job. It would be beneficial for longitudinal research to be undertaken to evaluate and monitor the effectiveness of such interventions and policies for the DV/SV workforce over time.

### Implications for Policy and Practice

The results of this review could help to provide information to specialist DV/SV organizations regarding which factors are important in the ProQOL of DV/SV advocates. Developing a comprehensive understanding of which factors could promote the positive impacts of DV/SV work, and which could mitigate the negative, is an important part of this process. The findings could be used to help inform targeted interventions such as the provision of specialist training, which aims to reduce risk factors and promote protective factors. As the review highlighted younger age as a risk factor for this workforce, organizations could provide interventions that aim to educate younger advocates on the potential impacts of the work (an identified protective factor—Campbell, 2008). For example, the delivery of training in relation to the impact, early signs and symptoms of CF, burnout, STS, and VT would be highly beneficial. In addition, building in opportunities for these advocates to access appropriate support (whether this be via self-care workshops or via peer support opportunities amongst colleagues). Given that communication competence was highlighted as a protective factor, findings such as this could be used to inform recruitment strategies. For example, DV/SV organizations could utilize questions or tasks focused on communication skills to assess potential advocates.

Finally, the findings indicate that steps should be taken to address unhealthy organizational cultures amongst some DV/SV organizations including a shift in individualistic attitudes toward well-being. Organizations must ensure that the burden of self-care is not placed solely on DV/SV advocates by providing effective responses to CF, burnout, and STS, etc. This includes developing policies which relate specifically to the negative impacts of the work and aim to protect/promote the well-being of staff members.

## Conclusion

In conclusion, this review demonstrated that well-being in DV/SV advocates is complex and an array of factors have the potential to influence their ProQOL. For DV/SV advocates to perform their roles effectively, it is imperative that they are supported to cope with the occupational stressors associated with this line of work. It is hoped that the findings of this review will be used to inform practical recommendations for specialist DV/SV organizations with the overall aim of improving advocates’ ProQOL and subsequently the quality and consistency of the services provided to victim/survivors.

### Critical Findings

- The ProQOL of DV/SV advocates is complex and dependent on a variety of risk and protective factors.
- For many of the factors identified, there was a lack of consistency regarding their impact across studies. For example, an advocate’s length of experience in the sector, whether they have experienced DV/SV themselves, and their level of client exposure.
- The importance of several new risk and protective factors for this workforce were identified including occupational stigma, communication skills, belief in a just world, exposure to microaggressions, and unhealthy organizational cultures.

### Implications of the Review for Practice, Policy, and Research

Implications for practice and policy:

- The findings of this review could be used to inform targeted interventions and the development of preventative policies and procedures for the DV/SV workforce.
- Unhealthy organizational cultures must be addressed, including a shift in individualistic attitudes toward well-being. Steps should be taken to ensure that the burden of self-care is not placed solely on DV/SV advocates.
- The specific needs and contributions of survivor advocates should be recognized, and where appropriate, additional support implemented.
- Many of the risk factors identified within the review (low pay, scarce resources, understaffing, etc.) demonstrate the need for additional funding in the sector. This would reduce some of the stressors faced by DV/SV advocates (e.g., by enabling organizations to employ more staff and ensuring advocates have sufficient resources to perform their role effectively).

Implications for future research:

- Existing research is more heavily focused on organizational factors. Future research should explore the role that personal strengths play in the well-being of those employed within the DV/SV sector.
- There is a need for clearer, more consistent, terminology, definitions, and measures used to assess well-being in this workforce.
- Future studies should aim to include participants on long term sickness, phased return, or those who have left the field to capture a range of perspectives.
- More research using mixed-methods designs would be beneficial.
- Future research should adopt a positive psychological approach by focusing on which factors have the potential to promote the positive impact of the work, as well as buffer against the negative.
- Longitudinal research should be undertaken including studies which aim to evaluate and monitor the effectiveness of well-being interventions and policies for the DV/SV workforce over time.

## Supplemental Material

sj-docx-1-tva-10.1177_15248380231171187 – Supplemental material for The Professional Quality of Life of Domestic and Sexual Violence Advocates: A Systematic Review of Possible Risk and Protective Factors Supplemental material, sj-docx-1-tva-10.1177_15248380231171187 for The Professional Quality of Life of Domestic and Sexual Violence Advocates: A Systematic Review of Possible Risk and Protective Factors by Harriet Bromley, Sarah K. Davis, Blaire Morgan and Holly Taylor-Dunn in Trauma, Violence, & Abuse

## Notes

1 Mixed methods systematic reviews combine the findings of qualitative and quantitative studies within a single systematic review to address the same overlapping or complementary review questions (Harden, 2010).

## References

[bibr1-15248380231171187] *Babin E. A. Palazzolo K. E. Rivera K. D. (2012). Communication skills, social support, and burnout among advocates in a domestic violence agency. Journal of Applied Communication Research, 40(2), 147–166. 10.1080/00909882.2012.670257

[bibr2-15248380231171187] *Baird S. Jenkins S. R. (2003). Vicarious traumatization, secondary traumatic stress, and burnout in sexual assault and domestic violence agency staff. Violence and Victims, 18(1), 71–86. 10.1891/vivi.2003.18.1.71 12733620

[bibr3-15248380231171187] *Baker L. M. O’Brien K. M. Salahuddin N. M. (2007). Are shelter workers burned out?: An examination of stress, social support, and coping. Journal of Family Violence, 22(6), 465–474. 10.1007/s10896-007-9103-1

[bibr4-15248380231171187] *Behounek E. K. (2011). “No help for the weary.” An ethnographic examination of factors impacting burnout among domestic violence and sexual assault advocates [Master’s thesis, University of Tennessee]. http://opensiuc.lib.siu.edu/gs_rp/50

[bibr5-15248380231171187] *Bemiller M. Williams L. S. (2011). The role of adaptation in advocate burnout: A case of good soldiering. Violence Against Women, 17(1), 89–110. 10.1177/1077801210393923 21199811

[bibr6-15248380231171187] Ben-Porat A. Itzhaky H. (2009). Implications of treating family violence for the therapist: Secondary traumatization, vicarious traumatization, and growth. Journal of Family Violence, 24(7), 507–515.

[bibr7-15248380231171187] Bishop S. Schmidt G. (2011). Vicarious traumatization and transition house workers in remote, northern British Columbia communities. Rural Society, 21(1), 65–73. 10.5172/rsj.2011.21.1.65

[bibr8-15248380231171187] Bozga A. McDowall A. Brown J. (2020). “Little red sandals”: Female police officers’ lived experience of investigating sexual violence. Policing, 44(1), 32–48. 10.1108/PIJPSM-02-2020-0029

[bibr9-15248380231171187] Brend D. M. Krane J. Saunders S. (2019). Exposure to trauma in intimate partner violence human service work: A scoping review. Traumatology, 26(1), 127–136. 10.1037/trm0000199

[bibr10-15248380231171187] *Brend D. M. MacIntosh H. B. (2021). Mentalizing as mechanism: An interpretive phenomenological analysis of workplace social support in intimate partner violence practice. Smith College Studies in Social Work, 91(1), 29–54. 10.1080/00377317.2020.1859432

[bibr11-15248380231171187] *Brown C. O’Brien K. M. (1998). Understanding stress and burnout in shelter workers. Professional Psychology: Research and Practice, 29(4), 383–385.

[bibr12-15248380231171187] *Campbell K. M. (2008). An analytical understanding of administrative practices minimizing vicarious traumatization in domestic violence organizations in Florida [Doctoral dissertation, University of Central Florida]. https://stars.library.ucf.edu/etd/3786/

[bibr13-15248380231171187] Clemans S. E. (2004). Life changing: The experience of rape-crisis work. Affilia – Journal of Women and Social Work, 19(2), 146–159. 10.1177/0886109903262758

[bibr14-15248380231171187] Critical Appraisal Skills Programme (2018). CASP qualitative checklist. https://casp-uk.net/wp-content/uploads/2018/03/CASP-Qualitative-Checklist-2018_fillable_form.pdf

[bibr15-15248380231171187] Crivatu I. M. Horvath M. A. H. Massey K. (2021). The impacts of working with victims of sexual violence: A rapid evidence assessment. Trauma, Violence, & Abuse, 24(1), 56–71. 10.1177/15248380211016024 PMC9660261 34000946

[bibr16-15248380231171187] Cunningham M. (2003). Impact of trauma work on social work clinicians: Empirical findings. Social Work, 48(4), 451–459. 10.1093/sw/48.4.451 14620102

[bibr17-15248380231171187] *Dekel R. Peled E. (2000). Staff burnout in Israeli battered women’s shelters. Journal of Social Service Research, 26(3), 65–76. 10.1300/J079v26n03_04

[bibr18-15248380231171187] Downes M. J. Brennan M. L. Williams H. C. Dean R. S. (2016). Development of a critical appraisal tool to assess the quality of cross-sectional studies (AXIS). BMJ Open, 6(12), 1–7. 10.1136/bmjopen-2016-011458 PMC5168618 27932337

[bibr19-15248380231171187] *Dworkin E. R. Sorell N. R. Allen N. E. (2016). Individual-and setting-level correlates of secondary traumatic stress in rape crisis center staff. Journal of Interpersonal Violence, 31(4), 743–752. 10.1177/0886260514556111 25381285

[bibr20-15248380231171187] *Fedele K. M. (2018). An investigation of factors impacting vicarious traumatization and vicarious posttraumatic growth in crisis workers: Vicarious exposure to trauma, feminist beliefs and feminist self-labelling [Doctoral dissertation, University of Akron]. https://etd.ohiolink.edu/apexprod/rws_olink/r/1501/10?clear=10&p10_accession_num=akron1519564198322496

[bibr21-15248380231171187] Figley C. R. (1995). Compassion fatigue: Toward a new understanding of the costs of caring. In Stamm B. H. (Ed.), Secondary traumatic stress: Self-care issues for clinicians, researchers, and educators (pp. 3–28). The Sidran Press.

[bibr22-15248380231171187] *Flatter K. J. (2000). The relationship between burnout and personality dimensions in domestic violence staff [Master’s thesis, Eastern Illinois University]. https://thekeep.eiu.edu/theses/1585

[bibr23-15248380231171187] *Frey L. L. Beesley D. Abbott D. Kendrick E. (2017). Vicarious resilience in sexual assault and domestic violence advocates. Psychological Trauma: Theory, Research, Practice, and Policy, 9(1), 44–51. 10.1037/tra0000159 27268097

[bibr24-15248380231171187] *Ganz J. (2015). Contested titles: Gendered violence victim advocacy and negotiating occupational stigma in social interactions [Doctoral thesis, Bowling Green State University]. https://scholarworks.bgsu.edu/acs_diss/74

[bibr25-15248380231171187] Giménez Lozano J. M. Martínez Ramón J. P. Morales Rodríguez F. M . (2021). Doctors and nurses: A systematic review of the risk and protective factors in workplace violence and burnout. International Journal of Environmental Research and Public Health, 18(6), 1–19. 10.3390/ijerph18063280 PMC8004742 33810020

[bibr26-15248380231171187] Hague G. (2021). The movement against domestic violence: Celebrating our history. https://www.womensaid.org.uk/movement-against-domestic-violence-celebrating-our-history/

[bibr27-15248380231171187] Harden A. (2010). Mixed-methods systematic reviews: Integrating quantitative and qualitative findings (Technical brief no. 25). National Center for the Dissemination of Disability Research (NCDDR). https://ktdrr.org/ktlibrary/articles_pubs/ncddrwork/focus/focus25/Focus25.pdf

[bibr28-15248380231171187] Hernández P. Gangsei D. Engstrom D. (2007). Vicarious resilience: A new concept in work with those who survive trauma. Family Process, 46(2), 229–241. 17593887 10.1111/j.1545-5300.2007.00206.x

[bibr29-15248380231171187] *Horvath M. A. H. Massey K. Essafi S. Majeed-Ariss R. (2020). Minimising trauma in staff at a sexual assault referral centre: What and who is needed? Journal of Forensic and Legal Medicine, 74, 102029. 10.1016/j.jflm.2020.102029 32759023

[bibr30-15248380231171187] *Horvath M. A. H. Rose H. Dalton T. Massey K. Matthews K. (2021). Independent Sexual Violence Advisers (ISVAs ) in England, Wales and Northern Ireland: A study of impacts, effects, coping mechanisms and effective support systems for people working as ISVAs and ISVA Managers (Project report). Middlesex University and Canterbury Christ Church University.

[bibr31-15248380231171187] Howarth E. Stimpson L. Barran D. Robinson A. (2009). Safety in numbers: A multisite evaluation of independent domestic violence advisor services. The Henry Smith Charity. https://safelives.org.uk/sites/default/files/resources/Safety_in_Numbers_full_report.pdf

[bibr32-15248380231171187] Jirek S. L. (2015). Soul pain: The hidden toll of working with survivors of physical and sexual Violence. SAGE Open, 5(3), 1–13. 10.1177/2158244015597905

[bibr33-15248380231171187] *Jirek S. L. (2020). Ineffective organizational responses to workers’ secondary traumatic stress: A case study of the effects of an unhealthy organizational culture. Human Service Organizations Management, Leadership and Governance, 44(3), 210–228. 10.1080/23303131.2020.1722302

[bibr34-15248380231171187] *Jury A. Thorburn N. Weatherall R. (2018). Workers’ constructions of the “good” and “bad” advocate in a domestic violence agency. Human Service Organizations: Management, Leadership & Governance, 42(3), 318–326. 10.1080/23303131.2018.1457583

[bibr35-15248380231171187] *Kreinath R. S. (2019). Secondary and vicarious traumatization among domestic violence shelter staff [Master’s thesis, Wichita State University]. http://hdl.handle.net/10057/16386

[bibr36-15248380231171187] *Kulkarni S. Bell H. Hartman J. L. Herman-Smith R. L. (2013). Exploring individual and organizational factors contributing to compassion satisfaction, secondary traumatic stress, and burnout in domestic violence service providers. Journal of the Society for Social Work and Research, 4(2), 114–130. 10.5243/jsswr.2013.8

[bibr37-15248380231171187] Kyron M. J. Rees C. S. Lawrence D. Carleton R. N. McEvoy P. M. (2021). Prospective risk and protective factors for psychopathology and wellbeing in civilian emergency services personnel: A systematic review. Journal of Affective Disorders, 281, 517–532. 10.1016/j.jad.2020.12.021 33388463

[bibr38-15248380231171187] Lee J. H. Nam S. K. Kim A. R. Kim B. Lee M. Y. Lee S. M. (2013). Resilience: A meta-analytic approach. Journal of Counseling and Development, 91(3), 269–279. 10.1002/j.1556-6676.2013.00095.x

[bibr39-15248380231171187] Lerner M. J. (1980). The belief in a just world. Springer US.

[bibr40-15248380231171187] Martínez-Martí M. L. Ruch W. (2017). Character strengths predict resilience over and above positive affect, self-efficacy, optimism, social support, self-esteem, and life satisfaction. Journal of Positive Psychology, 12(2), 110–119. 10.1080/17439760.2016.1163403

[bibr41-15248380231171187] Maslach C. (1982). Burnout: The cost of caring. Prentice-Hall, Inc.

[bibr42-15248380231171187] *Massey K. Horvath M. A. H. H. Essafi S. Majeed-Ariss R. (2019). Staff experiences of working in a Sexual Assault Referral Centre: The impacts and emotional tolls of working with traumatised people. Journal of Forensic Psychiatry & Psychology, 30(4), 686–705. 10.1080/14789949.2019.1605615

[bibr43-15248380231171187] Meadors P. Lamson A. Swanson M. White M. Sira N. (2010). Secondary traumatization in pediatric healthcare providers: Compassion fatigue, burnout and secondary traumatic stress. OMEGA – Journal of Death and Dying, 60(2), 103–128. 10.2190/OM.60.2.a 20222232

[bibr44-15248380231171187] *Merchant L. V. Whiting J. B. (2015). Challenges and retention of domestic violence shelter advocates: A grounded theory. Journal of Family Violence, 30(4), 467–478. 10.1007/s10896-015-9685-y

[bibr45-15248380231171187] Mihelicova M. Wegrzyn A. Brown M. Greeson M. R. (2019). Stressors of rape crisis work from the perspectives of advocates with and without sexual assault victimization history. Journal of Interpersonal Violence, 36(19/20), 1–24. 10.1177/0886260519876715 31542983

[bibr46-15248380231171187] *Molloy E. (2019). An exploration of social care workers experiences of emotional labour and professional burnout in domestic violence refuges professional burnout in domestic violence refuges. Journal of Social Care, 2(1), 1–23.

[bibr47-15248380231171187] Molnar B. E. Meeker S. A. Manners K. Tieszen L. Kalergis K. Fine J. E. Hallinan S. Wolfe J. D. Wells M. K. (2020). Vicarious traumatization among child welfare and child protection professionals: A systematic review. Child Abuse & Neglect, 110, 1–15. http://10.0.3.248/j.chiabu.2020.104679 10.1016/j.chiabu.2020.104679 32826062

[bibr48-15248380231171187] Page M. J. Moher D. Bossuyt P. M. Boutron I. Hoffmann T. C. Mulrow C. D. Shamseer L. Tetzlaff J. M. Akl E. A. Brennan S. E. (2021). PRISMA 2020 explanation and elaboration: Updated guidance and exemplars for reporting systematic reviews. BMJ, 372(160), 1–36. 10.1136/bmj.n160 PMC8005925 33781993

[bibr49-15248380231171187] Pearlman L. A. Caringi J. (2009). Vicarious traumatization and complex trauma. In Courtois C. A. Ford J. D. (Eds.), Complex traumatic stress disorders: An evidence-based clinician’s guide. Guilford Press.

[bibr50-15248380231171187] Rizkalla N. Zeevi-Barkay M. Segal S. P. (2021). Rape crisis counseling: Trauma contagion and supervision. Journal of Interpersonal Violence, 36(1/2), 1–24. 10.0.4.153/0886260517736877 29294964

[bibr51-15248380231171187] Schutte N. S. Malouff J. M. (2019). The impact of signature character strengths interventions: A meta-analysis. Journal of Happiness Studies, 20(4), 1179–1196. 10.1007/s10902-018-9990-2

[bibr52-15248380231171187] Silveira F. S. Boyer W. (2015). Vicarious resilience in counselors of child and youth victims of interpersonal trauma. Qualitative Health Research, 25(4), 513–526. 10.1177/1049732314552284 25246327

[bibr53-15248380231171187] *Slattery S. M. Goodman L. A. (2009). Secondary traumatic stress among domestic violence advocates: Workplace risk and protective factors. Violence Against Women, 15(11), 1358–1379. 10.1177/1077801209347469 19809098

[bibr54-15248380231171187] Stamm B. H. (2010). The concise ProQOL manual (2nd ed.) ProQOL.org https://ProQOL.org.

[bibr55-15248380231171187] Steel N. Blakeborough L. Nicholas S. (2011). Supporting high-risk victims of domestic violence: A review of Multi-Agency Risk Assessment Conferences (MARACs) (Home Office Research Report 55). Home Office.

[bibr56-15248380231171187] Stern C. Lizarondo L. Carrier J. Godfrey C. Rieger K. Salmond S. Apostolo J. Kirkpatrick P. Loveday H. (2020). Methodological guidance for the conduct of mixed methods systematic reviews. JBI Evidence Synthesis, 18(10), 2108–2118. 10.11124/JBISRIR-D-19-00169 32813460

[bibr57-15248380231171187] Szczygiel D. D. Mikolajczak M. (2018). Emotional intelligence buffers the effects of negative emotions on job burnout in nursing. Frontiers in Psychology, 9, 2649. 10.3389/fpsyg.2018.02649 30627113 PMC6309155

[bibr58-15248380231171187] *Taylor A. K. Gregory A. Feder G. Williamson E. (2019). ‘We’re all wounded healers’: A qualitative study to explore the well-being and needs of helpline workers supporting survivors of domestic violence and abuse. Health & Social Care in the Community, 27(4), 856–862. 10.1111/hsc.12699 30592098

[bibr59-15248380231171187] *Ullman S. E. Townsend S. M. (2007). Barriers to working with sexual assault survivors: A qualitative study of rape crisis center workers. Violence Against Women, 13(4), 412–443. 10.1177/1077801207299191 17420518

[bibr60-15248380231171187] *Voth Schrag R. J. Wood L. G. Wachter K. Kulkarni S . (2021). Compassion fatigue among the intimate partner violence and sexual assault workforce: Enhancing organizational practice. Violence Against Women, 28(1), 277–297. 10.1177/1077801220988351 33596785

[bibr61-15248380231171187] *Wachter K. Schrag R. V. Wood L. (2020). Coping behaviors mediate associations between occupational factors and compassion satisfaction among the intimate partner violence and sexual assault workforce. Journal of Family Violence, 35(2), 143–154. 10.1007/s10896-019-00072-0 32435084 PMC7223839

[bibr62-15248380231171187] Wasco S. M. Campbell R. (2002). Emotional reactions of rape victim advocates: A multiple case study of anger and fear. Psychology of Women Quarterly, 26(2), 120–130. 10.1111/1471-6402.00050

[bibr63-15248380231171187] *Wasco S. M. Campbell R. Clark M. (2002). A multiple case study of rape victim advocates’ self-care routines: The influence of organizational context. American Journal of Community Psychology, 30(5), 731–760. 10.1023/A:1016377416597 12188058

[bibr64-15248380231171187] *Wilson J. M. Goodman L. A. (2021). “A community of survivors”: A grounded theory of organizational support for survivor-advocates in domestic violence agencies. Violence Against Women, 27(14), 2664–2686. 10.1177/1077801220981143 33529567

[bibr65-15248380231171187] Wood L. (2014). Domestic violence advocacy [Doctoral dissertation, Indiana University]. https://www.proquest.com/openview/d1f61b735fd337ac5a9b1339f85739ee/1?pq-origsite=gscholar&cbl=18750

[bibr66-15248380231171187] Wood L. (2017). “I look across from me and I see me” survivors as advocates in intimate partner violence agencies. Violence Against Women, 23(3), 309–329. 10.1177/1077801216641518 27094434

[bibr67-15248380231171187] Wood L. Clark D. Heffron L. C. Schrag R. V. (2020). Voluntary, survivor-centered advocacy in domestic violence agencies. Advances in Social Work, 20(1), 1–21.

