# Supplementary material for: The Professional Quality of Life of Domestic and Sexual Violence Advocates: A Systematic Review of Possible Risk and Protective Factors
Source: Trauma Violence Abuse. 2023 May 18;25(2):1113–28. doi: 10.1177/15248380231171187 (PMC10913293; doi:10.1177/15248380231171187)
Supplement: sj-docx-1-tva-10.1177_15248380231171187 – Supplemental material for The Professional Quality of Life of Domestic and Sexual Violence Advocates: A Systematic Review of Possible Risk and Protective Factors [file sj-docx-1-tva-10.1177_15248380231171187.docx]

**Supplementary Material**

**Systematic Review Search Terms Table**

| **Table A1**. |  |
| --- | --- |
| *Key Words used in the Academic Databases Search Strategy for the Systematic Review* | |
| Professional role/Work setting | Terms related to Professional Quality of Life |
| “Independent sexual violence*” OR  “Independent domestic violence*” OR  “Rape crisis cent*” OR  “Sexual assault referral cent*” OR  “Shelter advocates” OR  “Shelter workers” OR  “Refuge workers” OR  “Helpline workers” OR  “Domestic violence age*” OR  “Domestic violence advo*” OR  “Domestic violence service providers” OR  “Sexual assault workforce” OR  “Rape victim advocates” OR  “Gender-based violence advocates” OR  “Sexual violence pr*” OR  “Domestic violence pr*” OR  “Intimate partner violence pract*” | “Compassion fatigue” OR  “Vicarious trauma” OR  “Secondary traumatic stress” OR  “Burnout” OR  “Trauma contagion” OR  “Compassion satisfaction” OR  “Resilience” OR  “Coping” OR  “Adaptation” OR  “Stressors” OR  “Risk factors” OR  “Protective factors” OR  “Individual factors” OR  “Organi?ational factors” |

**Detailed Quality Appraisal Tables**

| **Table B1.**  *Details of Quality Appraisals for the Quantitative Studies included in the Systematic Review.* | | | | | | | | | | | | | | | | | | | | |  |
| --- | --- | --- | --- | --- | --- | --- | --- | --- | --- | --- | --- | --- | --- | --- | --- | --- | --- | --- | --- | --- | --- |
| Study Author (Year) | 1 | 2 | 3 | 4 | 5 | 6 | 7 | 8 | 9 | 10 | 11 | 12 | 13 | 14 | 15 | 16 | 17 | 18 | 19 | 20 | Quality Score |
| Babin et al., (2012) | Y | Y | N | Y | Y | N | N/S | Y | N | Y | Y | Y | N/S | N/S | N | Y | Y | Y | N/S | Y | Moderate |
| Baird & Jenkins (2003) | N | Y | N | Y | Y | N | N/S | Y | Y | Y | Y | Y | N/S | N/S | N | Y | N | N | N/S | Y | Moderate |
| Baker et al., (2007) | N | Y | N | Y | Y | N | N/S | Y | N | Y | Y | Y | N/S | N/S | Y | Y | Y | Y | N/S | N/S | Moderate |
| Bemiller & Williams (2011) | N | Y | N | Y | Y | N | N/S | Y | N | Y | Y | Y | N/S | N/S | Y | Y | Y | N | N | N/S | Moderate |
| Brown & O’Brien (1998) | Y | Y | N | Y | Y | N | N/S | Y | Y | N | N | N | N/S | N/S | N/S | N | Y | N | N/S | N/S | Moderate |
| Campbell (2008) | Y | Y | Y | Y | Y | N | N/S | Y | Y | Y | Y | Y | N/S | N/S | Y | Y | Y | Y | N/S | Y | High |
| Dekel & Peled (2000) | Y | Y | Y | Y | Y | N | N/S | Y | N | Y | N | Y | N/S | N/S | Y | Y | Y | Y | N/S | N/S | Moderate |
| Dworkin et al., (2016) | Y | Y | N | Y | Y | N | N/S | Y | N | Y | N | Y | N/S | N/S | N | Y | N | Y | N | Y | Moderate |
| Fedele (2018) | Y | Y | Y | Y | Y | N | N/S | Y | Y | Y | Y | Y | N/S | N/S | Y | Y | Y | Y | N/S | Y | High |
| Flatter (2000) | Y | Y | N | Y | Y | N | N/S | Y | Y | Y | Y | Y | N/S | N/S | Y | Y | Y | Y | N/S | Y | High |
| Frey et al., (2017) | Y | Y | N | Y | Y | N | N/S | Y | Y | Y | Y | Y | N/S | N/S | Y | Y | Y | Y | N/S | Y | High |
| Ganz (2015)  Quantitative phase | Y | Y | N | Y | Y | N | N/S | Y | N | Y | Y | Y | N/S | N/S | Y | Y | Y | N | N/S | Y | Moderate |
| Horvath et al., (2021) | Y | Y | N | Y | Y | N | N/S | Y | Y | Y | Y | Y | N/S | N/S | Y | Y | Y | Y | N/S | Y | High |
| Kulkarni et al., (2013) | Y | Y | Y | Y | Y | N | N/S | Y | N | Y | Y | Y | N/S | Y | Y | Y | Y | Y | N | Y | High |
| Slattery & Goodman (2009) | Y | Y | N | Y | Y | N | N/S | Y | Y | Y | Y | Y | N/S | Y | Y | Y | Y | Y | N | Y | High |
| Voth Schrag et al., (2021) | Y | Y | N | Y | Y | N | N/S | Y | Y | Y | Y | Y | N/S | N/S | Y | Y | Y | Y | N | Y | High |
| Wachter et al., (2020) | Y | Y | N | Y | Y | N | N/S | Y | Y | Y | Y | Y | N/S | N/S | N | Y | Y | Y | N | Y | High |

*Note*. Studies appraised using The Appraisal Tool for Cross-Sectional Studies (AXIS; Downes et al., 2016). N/S indicates ‘not stated’. Quality score ranged 0-20 and was based upon total items fully satisfied: Low (0-6), Moderate (7-13), High (14-20) quality.

**Introduction**

1. Were the aims/objectives of the study clear?

**Methods**

1. Was the study design appropriate for the stated aim(s)?
2. Was the sample size justified?
3. Was the target/reference population clearly defined? (Is it clear who the research was about?)
4. Was the sample frame taken from an appropriate population base so that it closely represented the target/reference population under investigation?
5. Was the selection process likely to select subjects/participants that were representative of the target/reference population under investigation?
6. Were measures undertaken to address and categorise non-responders?
7. Were the risk factor and outcome variables measured appropriate to the aims of the study?
8. Were the risk factor and outcome variables measured correctly using instruments/measurements that had been trialled, piloted or published previously?
9. Is it clear what was used to determined statistical significance and/or precision estimates? (e.g. p-values, confidence intervals)
10. Were the methods (including statistical methods) sufficiently described to enable them to be repeated?

**Results**

1. Were the basic data adequately described?
2. Does the response rate raise concerns about non-response bias? (R)
3. If appropriate, was information about non-responders described?
4. Were the results internally consistent?
5. Were the results presented for all the analyses described in the methods?

**Discussion**

1. Were the authors' discussions and conclusions justified by the results?
2. Were the limitations of the study discussed?

**Other**

1. Were there any funding sources or conflicts of interest that may affect the authors’ interpretation of the results? (R)
2. Was ethical approval or consent of participants attained?

| **Table B2.**  *Details of Quality Appraisals for the Qualitative Studies included in the Systematic Review.* | | | | | | | | | | | |
| --- | --- | --- | --- | --- | --- | --- | --- | --- | --- | --- | --- |
| Study Author (Year) | 1 | 2 | 3 | 4 | 5 | 6 | 7 | 8 | 9 | 10 | Quality Appraisal |
| Behounek (2011) | Y | Y | Y | Y | C/T | N | Y | N | Y | Y | Moderate |
| Brend & MacIntosh (2021) | Y | Y | Y | Y | Y | N | Y | Y | Y | Y | High |
| Horvath et al., (2020) | Y | Y | Y | Y | Y | N | Y | N | Y | Y | High |
| Jirek (2020) | Y | Y | Y | Y | Y | N | Y | Y | Y | Y | High |
| Jury et al., (2018) | Y | Y | Y | Y | Y | N | N | N | Y | Y | Moderate |
| Kreinath (2019) | Y | Y | Y | Y | Y | N | Y | Y | Y | Y | High |
| Massey et al., (2019) | Y | Y | Y | Y | Y | Y | Y | Y | Y | Y | High |
| Merchant & Whiting (2015) | Y | Y | Y | Y | Y | Y | N | Y | Y | Y | High |
| Molloy (2019) | N | Y | Y | C/T | C/T | N | Y | N | Y | Y | Weak |
| Taylor et al., (2019) | Y | Y | Y | Y | Y | N | Y | N | Y | Y | High |
| Ullman & Townsend (2007) | Y | Y | Y | Y | Y | N | N | Y | Y | Y | High |
| Wasco et al., (2002) | Y | Y | Y | Y | Y | N | C/T | Y | Y | Y | High |
| Wilson & Goodman (2021) | Y | Y | Y | Y | Y | Y | N | Y | Y | Y | High |

*Note*. Studies appraised using the CASP quality assessment tool for qualitative studies (Critical Appraisal Skills Programme, 2018). C/T indicates ‘can’t tell’.

1. Was there a clear statement of the aims of the research?
2. Is a qualitative methodology appropriate?
3. Was the research design appropriate to address the aims of the research?
4. Was the recruitment strategy appropriate to the aims of the research?
5. Was the data collected in a way that addressed the research issue?
6. Has the relationship between researcher and participants been adequately considered?
7. Have ethical issues been taken into consideration?
8. Was the data analysis sufficiently rigorous?
9. Is there a clear statement of findings?
10. Is the research valuable?

**Systematic Review Supplementary Tables**

| **Table C1.**  *Study Descriptions of the 17 Quantitative Studies included in the Systematic Review of Risk and Protective Factors in the Well-being of Domestic and Sexual Violence Advocates.* | | | | | | | |
| --- | --- | --- | --- | --- | --- | --- | --- |
| Reference | Study design | Country of origin | Sample characteristics | Aspect of well-being | Well-being measure | Relevant findings | Quality appraisal |
| Babin et al., (2012) | Cross-sectional | United States | Shelter staff, board members & shelter volunteers from one DV agency N = 69 (87.5% female, age range 21-60 years). | Burnout | MBI | Risk factors: Communication anxiety.  Protective factors: Communication competence, Perceived informational support at work, Perceived emotional support at work. | Moderate |
| Baird & Jenkins (2003) | Cross-sectional | United States | Paid & volunteer roles – crisis worker, hotline worker, caseworker, case manager, counsellor, therapist, psychologist, intern, supervisor, director, and educator from 8 DV and/or SV agencies N = 101 (96 females, 4 males & 1 other, age range 21-65 years). | Burnout, CF,  VT | MBI  CFST  TSI-BSL | Risk factors: Younger age.  Protective factors: Years of education, Years of experience.  No association: Client exposure. | Moderate |
| Baker et al., (2007) | Cross-sectional | United States | Shelter workers from 9 women’s crisis centres (83% DV shelter, 17% homeless shelter where high % of the women had experienced DV) N = 123 (100% female, mean age 36.97 years). | Burnout | MBI | Risk factors: High levels of time pressure, Low levels of self-efficacy for being productive at work.  Protective factors: High levels of time pressure, High levels of self-efficacy for dealing with stressors at work.  No association: Workplace social support, Coping strategies. | Moderate |
| Bemiller & Williams (2011) | Cross-sectional | United States | Advocates employed at 30 DV and SV shelters N = 194 (99.5% female & 0.5% male, age range 21-60+ years). | Burnout | MBI | Risk factors: Hours worked, After hour responsibilities, Working with outside agencies, Office resources, Co-worker stress.  Protective factors: “Good soldiering” experience.  No association: Co-worker and supervisor support, Fair practices, Safety, Personal experience with victimization. | Moderate |
| Brown & O’Brien (1998) | Cross-sectional | United States | Shelter workers from 6 DV shelters N = 91 (89 female, 1 male & 1 gender not indicated, mean age 32.80 years). | Burnout | MBI | Risk factors: Job related stress (time pressure), Mental disengagement as a coping strategy.  Protective factors: Social support from supervisor, Social support from family and friends; Problem focused coping mechanisms. | Moderate |
| Campbell (2008) | Cross-sectional | United States | DV shelter staff who provide direct-care services or administrators who supervise direct-care staff N = 112 (99% female & 1% male, no age range provided). | VT | TABS | Protective factors: Knowledge of VT, Co-worker support, Supervision. | High |
| Dekel & Peled (2000) | Cross-sectional | Israel | Workers employed in Israeli battered women’s shelters N = 44 (100% female, age range 24-69 years). | Burnout | MBI | Risk factors: Hours worked, Client exposure.  Protective factors: Education, Experience.  No association: Social support. | Moderate |
| Dworkin et al., (2016) | Cross-sectional | United States | SV advocates, administrators, educators, counsellors & volunteers from 40 rape crisis centres N = 164 (gender not provided, age range 21-67 years). | STS | PCL-C | Risk factors: Younger age, History of SA, Higher client loads. | Moderate |
| Fedele (2018) | Cross-sectional | United States | Crisis workers the majority of whom were caseworkers/advocates based within a DV agency (42.5%) and rape crisis centres (15%) N = 149 (100% female, age range 19-70 years). | VT  VG | TSI-BSL  PTGI | Risk factors: Feminist beliefs, Feminist self-labelling.  Protective factors: Feminist beliefs.  No association: Degree of vicarious exposure to trauma. | High |
| Flatter (2000) | Cross-sectional | United States | DV staff from 10 DV agencies N = 69 (67 female, 1 male & 1 missing, mean age 39 years, no age range provided). | Burnout | MBI | Risk factors: Neuroticism, Younger age, Amount of hours spent per week in case management or staff meetings.  Protective factors: Job satisfaction, Workplace social support. | High |
| Frey et al., (2017) | Cross-sectional | United States | DV and/or SV advocates, crisis intervention, counsellors, shelter workers, medical advocacy N = 222 (212 female, 8 male & 2 genderqueer or fluid, age range 20-68 years). | CS  VPTG | Pro-QoL  VPTGI | Protective factors: History of trauma,  Peer relational quality, Organizational support, Experience in the sector. | High |
| Ganz (2015) | Mixed method: Cross sectional phase | United States | DV and/or SV advocates.  Qual: N = 21 (20 female & 1 male, age range 23-69 years).  Quan: N = 382 (348 female, 4 genderqueer, 3 male & 27 not reported, age range not provided). | Burnout | Adapted MBI | Risk factors: Occupational stigma, Number of weekly hours, Hours of training prior to employment.  Protective factors: Support for occupation, External social support, Age, Years in advocacy, self-care (professional boundary keeping). | Moderate |
| Horvath et al., (2021) | Cross-sectional | United Kingdom | ISVA’s and ISVA managers N = 121 (92.6% females & 7.4% males, age range 23-63 years). | VR  VT  Distress  PTSD | VRS  VTS  CORE-10  BRESLAU | Risk factors: High caseloads, Length of time working in an SV role.  Protective factors: Positive coping mechanisms (including spending time with family and friends), Belief in a just world. | High |
| Kulkarni et al., (2013) | Cross-sectional | United States | DV advocates, shelter workers, counsellors & outreach educators from numerous organizations N = 236 (95% women, age range 22-68 years). | CS  Burnout  STS | Pro-QOL | Risk factors: Higher workloads, Control over work environment, Time spent in leisure.  Protective factors: Years of experience, Supervision quality, Shared values, Self-care activities. | High |
| Slattery & Goodman (2009) | Cross-sectional | United States | DV advocates, counsellors, clinical or programme coordinators and directors from numerous organizations N = 148 (100% female, age range 19-65 years). | STS | PCL-S | Risk factors: History of abuse.  Protective factors: Support from co-workers, Quality of supervision, Shared power.  No association: Number of direct service hours. | High |
| Voth Schrag et al., (2021) | Cross-sectional | United States | DV & SV advocates (numerous roles but not specified) working with survivors and/or perpetrators N = 520 (no gender provided, age range 18-73 years). | CF | Pro-QOL | Risk factors: Recent life stress, Higher workload, Direct practice with survivors, Exposure to microaggressions.  Protective factors: Older age (even when controlling for years of experience), Satisfaction with supervision, Control over work environment, A sense of community,  Fairness, Shared values.  No association: History of trauma. | High |
| Wachter et al., (2020) | Cross-sectional | United States | Advocacy role that involved at least 50% of work time focused on DV/SV related issues, and/or employment where DV/SV were a central focus of the organizational mission N = 623 (92.9% female, 5.4% male & 1.8% another gender identity, age range 21-73 years). | CS | Pro-QOL | Risk factors: Higher workload.  Protective factors: Control over work environment, Meaningful rewards,  A sense of community, Fairness, Shared values, Frequency & range of coping behaviours, Job security. | High |

*Note.* MBI = Maslach Burnout Inventory; CF = Compassion Fatigue; VT = Vicarious Trauma; CFST = Compassion Fatigue Self-Test; TSI-BSL = Traumatic Stress Institute Belief Scale-Revision L; TABS = Trauma and Attachment Belief Scale; STS = Secondary Traumatic Stress; PCL-C = Posttraumatic Checklist–Civilian Version; PTGI = Post Traumatic Growth Inventory; CS = Compassion Satisfaction; VPTG = Vicarious Post Traumatic Growth; ProQoL = Professional Quality of Life; VPTGI = Vicarious Post Traumatic Growth Inventory; VR = Vicarious Resilience; PTSD = Post Traumatic Stress Disorder; VRS = Vicarious Resilience Scale; VTS = Vicarious Trauma Scale.

| **Table C2.**  *Study Descriptions of the 13 Qualitative Studies included in the Systematic Review of Risk and Protective Factors in the Well-being of Domestic and Sexual Violence Advocates.* | | | | | |
| --- | --- | --- | --- | --- | --- |
| Reference | Study design | Country of origin | Sample characteristics | Relevant findings | Quality appraisal |
| Behounek (2011) | Semi-structured interviews | United States | 3 former and 7 current domestic and sexual violence advocates N = 10 (100% female, age range 26-50 years). | Risk factors: History of abuse, Low pay, On-call shifts, Exposure to trauma, Collaboration issues, Stress management within organizations. | Moderate |
| Brend & MacIntosh (2021) | Semi-structured interviews | Canada | Social workers from 5 different not-for-profit organizations offering psychosocial services to people impacted by IPV N = 5 (gender not provided, age range 30-38 years). | Protective factors: Workplace social support. | High |
| Horvath et al., (2020) | Semi-structured interviews | United Kingdom | ISVA’s, crisis workers and forensic physicians from one SARC N = 16 (100% female, no ages provided). | Protective factors: Positive co-worker relationships, Supervision, Training  Coping skills (being reflexive). | High |
| Jirek (2020) | Case study | United States | Advocates, program coordinators & supervisors from one DV/SV agency N = 29 (100% female, age range 21-40+ years). | Risk factors: Unhealthy organizational culture (norms & collective expectations), Supervisors who failed to model effective self-care strategies, High workloads. | High |
| Jury et al., (2018) | Qualitative survey & semi-structured interviews | New Zealand | Advocates, volunteers & managers from one DV agency N = 111 (no gender or age range provided). | Risk factors: Unhealthy organizational culture (norms & collective expectations). | Moderate |
| Kreinath (2019) | Semi-structured interviews | United States | DV shelter staff, four of whom held administrative positions N = 15 (14 females & 1 male, age range 26-59 years). | Risk factors: Lack of support from supervisors and administrators, Poor work conditions (scarce resources).  Protective factors: Length of experience, Self-care and coping strategies (including support from partners/family/friends), Workplace social support. | High |
| Massey et al., (2019) | Semi-structured interviews | United Kingdom | ISVA’s, crisis workers and doctors N = 16 (100% female, no age range provided). | Risk factors: Out of hours work, Higher workloads.  Protective factors: Informal support from co-workers, Formal supervision, Coping strategies. | High |
| Merchant & Whiting (2015) | Semi-structured interviews | United States | Current and former shelter workers from 9 DV shelters N = 19 (17 female & 2 male, age range 23-61 years). | Protective factors: Supportive shelter culture. | High |
| Molloy (2019) | Semi-structured interviews | Ireland | Social care workers based in a DV refuge N = 4 (100% female, no age range provided). | Risk factors: Organizational support  Protective factors: Organizational support, Emotional boundaries, Self-awareness, Education, Experience. | Weak |
| Taylor et al., (2019) | Semi-structured interviews | United Kingdom | Helpline workers working for DV helplines N = 10 (100% female, age range 22-54 years). | Risk factors: Lack of training, Lack of regular supervision.  Protective factors: Self-care strategies. | High |
| Ullman & Townsend (2007) | Semi-structured interviews | United States | Current or former rape victim advocates from rape crisis centres N = 25 (100% female, age range 25-58 years). | Risk factors: Inadequate supervision, Lack of adequate pay, Rigid work demands (inflexibility), Lack of organizational support. | High |
| Wasco et al., (2002) | Semi-structured interviews | United States | Rape victim advocates N = 8 (gender not provided, age range 27-52 years). | Protective factors: Organizational support, Self-care routines. | High |
| Wilson & Goodman (2021) | Semi-structured interviews | United States | DV survivor-advocates from 9 DV agencies N = 12 (11 female & 1 gender nonconforming, age range 25-58 years). | Protective factors: An organization that encourages self-care, A sense of belonging, Shared values, Valuing survivors’ expertise. | High |
